# Rate of deep-vein thrombosis and pulmonary embolism during the care continuum in patients with acute ischemic stroke in the United States

**DOI:** 10.1186/1471-2377-13-17

**Published:** 2013-02-08

**Authors:** Alpesh N Amin, Jay Lin, Stephen Thompson, Daniel Wiederkehr

**Affiliations:** 1 Department of Medicine, University of California-Irvine, 101 The City Drive South, Building 26, Room 1005, ZC-4076H, Orange, CA, 92868, USA; 2 Novosys Health, Flemington, NJ, USA; 3 Sanofi U.S., Inc, Bridgewater, NJ, USA; 4 Pfizer Inc, New York, NY, USA

**Keywords:** Acute ischemic stroke, Care continuum, Deep-vein thrombosis, Pulmonary embolism, Thromboprophylaxis

## Abstract

**Background:**

Deep-vein thrombosis (DVT) and pulmonary embolism (PE) are frequent and life-threatening complications of ischemic stroke. We evaluated rates of symptomatic DVT/PE, and of in-hospital and post-discharge thromboprophylaxis in patients with acute ischemic stroke (AIS).

**Methods:**

In a retrospective US database analysis, data were extracted from the Premier Perspective™-i3 Pharma Informatics linked database for patients aged ≥18 years who were hospitalized for ischemic stroke from January 2005 to November 2007, and who had ≥6 months’ continuous plan enrollment prior to index hospitalization. Patients discharged to an acute-care facility or with atrial fibrillation were excluded. Prophylaxis was evaluated during index hospitalization and for 14 days’ post-discharge. DVT/PE rates were calculated during index hospitalization and up to 30 days post-discharge.

**Results:**

A total of 1524 patients were included; 46.1% received pharmacological and/or mechanical prophylaxis in-hospital (28.3%, 11.4% and 12.3% received unfractionated heparin, enoxaparin and mechanical prophylaxis, respectively). 6.4% of patients received outpatient pharmacological prophylaxis; warfarin was most frequently prescribed (5.9%). Total mean ± standard deviation length of index hospitalization was 3.0 ± 2.5 days. Mean prophylaxis duration in all patients was 0.9 ± 1.5 days in-hospital and 1.7 ± 6.9 days post-discharge. Symptomatic DVT/PE occurred in 25 patients overall (1.64%), with an inpatient rate of 0.98% and an outpatient rate of 0.66%.

**Conclusions:**

Approximately 1% of patients with AIS experienced symptomatic in-hospital and/or post-discharge DVT/PE. Although 46% received prophylaxis in-hospital, only 6% received prophylaxis in the outpatient setting. This highlights the need for sustained thromboprophylaxis prescribing across the continuum of care.

## Background

Deep-vein thrombosis (DVT) and pulmonary embolism (PE) are frequent complications of ischemic stroke [1,2]. There is a large variation in incidence among different clinical studies, with clinically confirmed DVT and PE in patients with ischemic stroke without thromboprophylaxis ranging from 1.0% to 5.2% and 0% to 5.6%, respectively [3]. PE is an important cause of mortality in patients after stroke; early studies indicated that PE accounted for up to a quarter of premature deaths in the absence of prophylaxis [4,5]. In a more recent large registry study of 13 440 patients with ischemic stroke by Heuschmann *et al.*, 0.4% of patients developed PE and nearly half (46.8%) of these patients died before hospital discharge [6].

Prophylaxis with low-molecular-weight heparins (LMWHs) and unfractionated heparin (UFH) reduces the risk of DVT in patients after acute ischemic stroke (AIS) [7-11]. Evidence-based guidelines from the American College of Chest Physicians (ACCP) recommend that AIS patients with restricted mobility receive LMWHs or UFH (Grade 1A) for the prevention of DVT/PE [12]. Although none of the LMWHs is indicated for DVT prophylaxis in ischemic stroke patients *per se*, these patients are often categorized as medical patients with reduced mobility—a group of patients for which dalteparin and enoxaparin are indicated. However, in real-world practice, many at-risk patients with ischemic stroke do not receive any prophylaxis [13-17]. In the Post-Stroke Rehabilitation Outcomes Project (PSROP), approximately a third of the 1161 at-risk patients had no documented orders for anticoagulants [16]. Even in those patients who do receive prophylaxis, it is often inappropriate in terms of type, dose, and/or duration [13-15], and this may limit its effectiveness at preventing DVT/PE. The worldwide ENDORSE (Epidemiologic International Day for the Evaluation of Patients at Risk for Venous Thromboembolism in the Acute Hospital Care Setting) study included 2423 patients with ischemic stroke. Only 47.1% of at-risk patients received any form of thromboprophylaxis and only 37.1% of patients received prophylaxis in-line with ACCP recommendations [13].

Patients with cerebrovascular disease spend on average 5.2 days in hospital [18]; therefore, outpatient prophylaxis may be required to enable the majority of patients to receive the 8 to 16 day regimens that were effective in clinical studies [7-11,19]. To understand current prescribing practices, further investigations are required on real-world use of thromboprophylaxis in patients with AIS, both in-hospital and also post-discharge where fewer data exist. There is also a need to assess actual DVT/PE rates in inpatient and outpatient settings to determine the current clinical burden associated with DVT/PE. The objective of this analysis was to evaluate symptomatic rates of DVT/PE events, and provision of prophylaxis for DVT throughout the continuum of care (in-hospital and post-discharge) in US patients with AIS.

## Methods

An observational, retrospective database analysis was performed on national managed care data. As such, this study was not governed by an Institutional Review Board. Data were extracted from the Premier Perspective™-i3 Pharma Informatics linked database, a large de-identified US hospital clinical and economic database developed for quality and utilization benchmarking. Claims data drawn from a large national health plan were cross-matched on the individual patient level for both in-hospital and post-discharge records. Discharge records were included in the analysis if patients met the following criteria: index hospitalization between January 2005 and November 2007; age ≥ 18 years at the time of index hospitalization; and ≥ 6 months’ continuous plan enrollment prior to the index hospitalization. The 6-month period prior to index admission was used to assess the presence of known risk factors for DVT/PE in the patient history through medical claims. Patients also had to be hospitalized for ischemic stroke as identified by *International Classification of Diseases, Ninth Revision, Clinical Modification* (ICD-9-CM) code searches (codes 430.x, 431.x, 433.x1, 434.x1, 435.x, 436, and 362.3). Patients were excluded if they had a length of hospital stay of 0 days or > 30 days, had missing/unknown gender or age data, or if they were diagnosed with atrial fibrillation. Patients were also excluded if they were discharged or transferred to an acute-care facility because outpatient prescriptions for anticoagulants or DVT/PE readmissions for these patients would not have been captured due to treatment in another facility.

Inpatient prophylaxis, mechanical and/or pharmacological, was captured via charge codes during hospitalization. Outpatient pharmacological prophylaxis was assessed as prescriptions during the 14-days prior to index admission and the 14-days following index discharge. Outpatient pharmacological prophylaxis was allowed to be initiated up to 14-days before the index admission to reflect that some patients might have received their anticoagulation prescriptions prior to pre-scheduled hospitalizations. Inpatient and outpatient pharmacological prophylaxis was identified via charge codes or pharmacy claims for UFH, LMWHs (enoxaparin, dalteparin, and tinzaparin), fondaparinux, and warfarin. Pharmacological agents were considered prophylactic if they were used at prophylaxis dosages and used prior to any venous thromboembolism (VTE) event. Mechanical prophylaxis was identified via charge codes for graduated compression stockings (GCS) and charge codes indicating the use of intermittent pneumatic compression devices and/or venous foot pumps. Outpatient use of mechanical prophylaxis was not captured due to over-the-counter availability. Combination prophylaxis usage was also analyzed, and was defined as use of more than one product/type of prophylaxis across the entire duration of the hospitalization or in the outpatient setting. The presence or absence of prophylaxis and the type used were calculated descriptively. The mean ± s.d. length of hospitalization, and the duration of inpatient and outpatient prophylaxis were also calculated; the prophylaxis duration was included as 0 for patients receiving no prophylaxis.

Patients were followed for up to 30-days after discharge in the analysis of symptomatic DVT/PE rates, and were censored administratively at December 2007 or plan disenrollment, whichever occurred first. Symptomatic DVT/PE events were defined as the first diagnosis according to ICD-9-CM codes and were categorized as index events (primary or secondary diagnosis during index admission), readmission events (primary or secondary diagnosis during a hospital admission following index hospitalization), or outpatient events (diagnosis in an outpatient setting accompanied by treatment with anticoagulant within 14 days of diagnosis).

## Results

**Figure 1:**
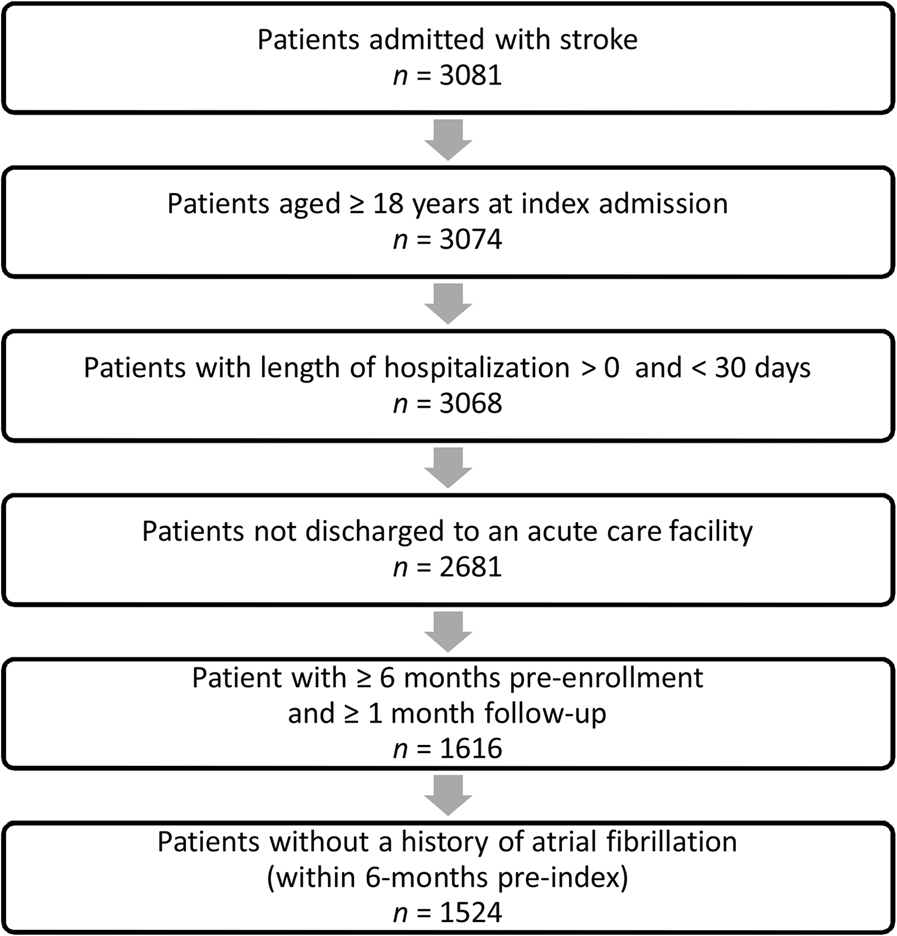
Flow diagram of patient inclusions.

A total of 1524 patients were included in the analysis (Figure 1), with a mean ± s.d. age of 62.2 ± 12.2 years (Table 1). Most patients were white (72.1%) and from the south of the US (52.6%). The majority of patients were commercially insured (91.1%) and were treated in urban hospitals (92.7%), without teaching status (65.0%).

**Table 1:** Summary of patient demographics and characteristics

***Characteristic***	***Stroke patients (*n** ***= 1524)***
Gender, *n* (%)	
Male	869 (57.02)
Female	655 (42.98)
Mean ± s.d. age, years	62.20 ± 12.23
Race, *n* (%)	
White	1098 (72.05)
Black	166 (10.89)
Hispanic	55 (3.61)
Other/unknown	205 (13.45)
Primary payer, *n* (%)	
Medicare	109 (7.15)
Medicaid	26 (1.71)
Commercial	1389 (91.14)
Geographical area, *n* (%)	
Northeast	96 (6.30)
Midwest	235 (15.42)
South	801 (52.56)
West	392 (25.72)
Urban location, *n* (%)	1413 (92.72)
Teaching hospital, *n* (%)	534 (35.04)

Less than half of the patients (46.1%) received any form of thromboprophylaxis during the index hospitalization. The most frequently prescribed pharmacological prophylactic agents were UFH (28.3%) and enoxaparin (11.4%) (Table 2). Other LMWHs and fondaparinux were prescribed very infrequently. One in ten patients received combination prophylaxis, and mechanical prophylaxis was received by approximately an eighth of all patients. Outpatient pharmacological prophylaxis was received by 6.4% of patients in the 14-day period post-discharge. The most commonly prescribed post-discharge prophylactic agent was warfarin (5.9%) (Table 2). Enoxaparin was the only other pharmacological agent prescribed in the outpatient setting (1.8%), most commonly in combination with warfarin (1.3%).

**Table 2:** Deep-vein thrombosis prophylaxis type in ischemic stroke patients during hospitalization and in the 14-day period post-discharge

	***Patients (*n** ***= 1524)***
**Index hospitalization, *n *(%)**	
Received any prophylaxis	703 (46.1)
Pharmacological prophylaxis	591 (38.8)
Unfractionated heparin	431 (28.3)
Enoxaparin	174 (11.4)
Dalteparin	1 (0.1)
Tinzaparin	0
Fondaparinux	1 (0.1)
Warfarin	0
Combination prophylaxis^a^	153 (10.0)
Mechanical prophylaxis	187 (12.3)
Graduated compression stockings	60 (3.9)
Non-graduated compression stockings	148 (9.7)
**Outpatient, *n *(%)**	
Received any prophylaxis	98 (6.4)
Pharmacological prophylaxis	98 (6.4)
Unfractionated heparin	0
Enoxaparin	27 (1.8)
Dalteparin	0
Tinzaparin	0
Fondaparinux	0
Warfarin	90 (5.9)
Combination pharmacological prophylaxis^b^	19 (1.3)

^a^Use of more than one product/type of prophylaxis across the entire duration of the hospitalization.

^b^Use of more than one anticoagulant in the outpatient setting.

The mean ± s.d. length of index hospitalization among all AIS patients was 3.0 ± 2.5 days (Table 3). The mean total duration of prophylaxis among all patients with AIS was 2.6 ± 7.1 days, with a mean of 0.9 ± 1.5 days in the inpatient setting and 1.7 ± 6.9 days in the outpatient setting. The mean ± s.d. total duration among patients who received in-hospital prophylaxis was 3.1 ± 6.5 days, with a mean duration of inpatient and outpatient prophylaxis of 1.9 ± 1.6 days and 1.3 ± 5.9 days, respectively (Table 3). Most patients who received prophylaxis (77.0%) started initial inpatient prophylaxis on the first day of hospitalization, with 16.1% of patients starting prophylaxis on the second day of hospitalization. Overall, 54.1% of patients only received prophylaxis on the day of hospital admission; 18.3% of patients received prophylaxis up to the second day of hospitalization.

**Table 3:** Duration of hospital stay and prophylaxis

	***Duration, days***							
	***All ischemic stroke patients (*n** ***= 1524)***		***Any prophylaxis patients (*n** ***= 776)***		***In-hospital prophylaxis patients (*n** ***= 703)***		***Outpatient prophylaxis patients (*n** ***= 98)***	
	***Mean ± s.d.***	***Median (range)***	***Mean ± s.d.***	***Median (range)***	***Mean ± s.d.***	***Median (range)***	***Mean ± s.d.***	***Median (range)***
Length of index hospital stay	3.0 ± 2.5	2 (1–27)	3.0 ± 2.7	2 (1–27)	2.7 ± 2.2	2 (1–13)	5.2 ± 4.2	4 (1–27)
Inpatient prophylaxis duration	0.9 ± 1.5	0 (0–12)	1.7 ± 1.7	1 (0–12)	1.9 ± 1.6	1 (1–12)	1.2 ± 2.2	0 (0–9)
Outpatient prophylaxis duration	1.7 ± 6.9	0 (0–42)	3.4 ± 9.4	0 (0–42)	1.3 ± 5.9	0 (0–42)	27.0 ± 7.3	30 (2–42)
Total prophylaxis duration	2.6 ± 7.1	1 (0–43)	5.1 ± 9.3	1 (0–43)	3.1 ± 6.5	1 (1–43)	28.2 ± 7.7	30 (3–43)

**Figure 2:**
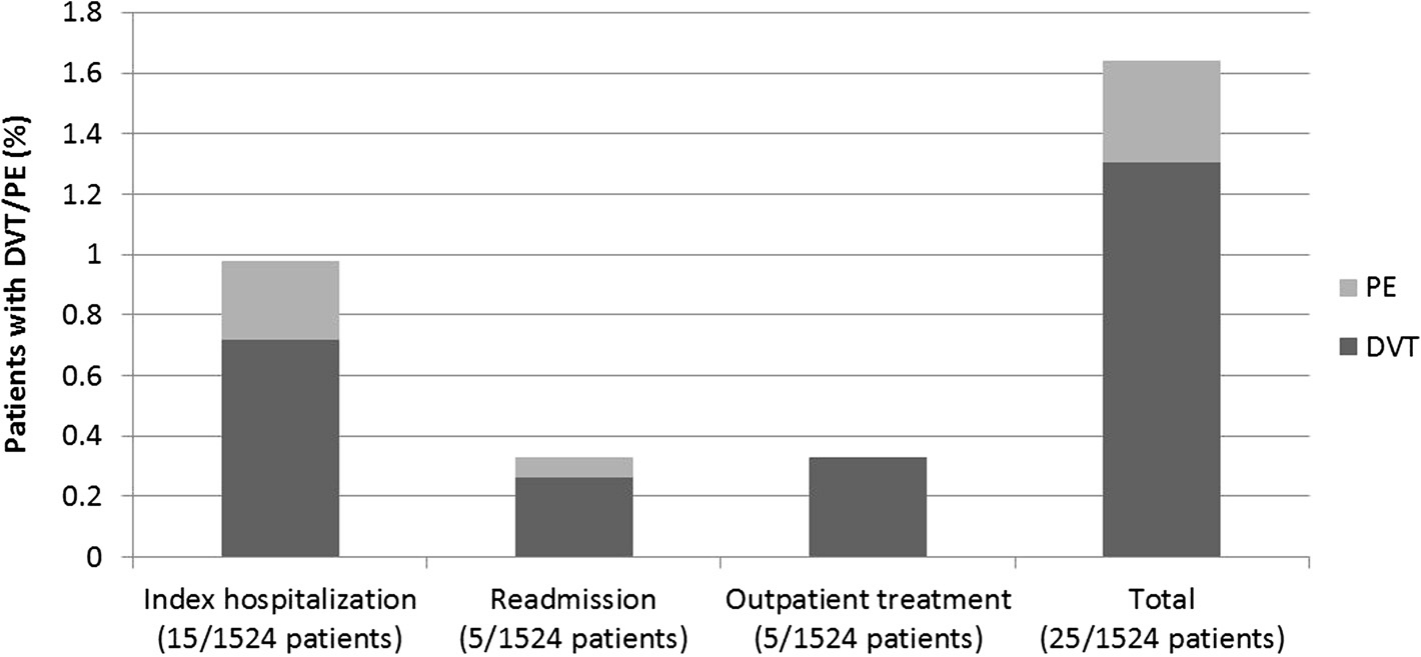
Deep-vein thrombosis (DVT)/pulmonary embolism (PE) rates during index hospitalization and rates in the outpatient setting in the 30-day period after discharge.

DVT/PE events occurred in 25 (1.64%) ischemic stroke patients in this study (Figure 2), which included 5 PE and 20 DVT events. Of these, 18 events occurred in patients who had received VTE prophylaxis and 7 events in patients who had not received VTE prophylaxis at index. DVT/PE events during index hospitalization occurred in 15 patients (0.98% of the total population; 60% of the total events). Ten patients developed DVT/PE in the outpatient setting (0.66%; 40% of the total events). Overall, 5 patients (0.33%) were readmitted for DVT/PE and 5 patients (0.33%) were treated for DVT/PE in the 30-day period post-discharge.

## Discussion

In the present real-world study, a total of 25 out of 1524 ischemic stroke patients developed symptomatic DVT/PE. The rate of PE of 0.33% is consistent with the PE rate of 0.4% obtained in the German Stroke Registers study by Heuschmann *et al.*[6]. In our study, 15 inpatients developed DVT/PE, highlighting the DVT/PE risk in-hospital. In addition, 10 patients were readmitted for DVT/PE or treated for DVT/PE in the outpatient setting, demonstrating that the risk of the occurrence of DVT/PE continues post-discharge. The risk of PE is thought to persist for up to 4 weeks after stroke; in patients who died in the second to fourth week after stroke, PE was the dominant cause of death as verified by autopsy [20].

In the present study, less than half of ischemic stroke patients received any form of prophylaxis in hospital and 6% received pharmacological prophylaxis post-discharge. These findings are consistent with several other studies demonstrating suboptimal prescribing practices in patients with ischemic stroke in-hospital [13-17]. The current study did not investigate the appropriateness of the prophylaxis provided but, given the results of other studies in medical patients, it is likely that prophylaxis was not in accordance with current guidelines in some patients. Few studies have investigated outpatient prophylaxis prescribing in patients with ischemic stroke. The present study analyzed anticoagulant prescriptions filled post-discharge, but this may include patients receiving an anticoagulant for reasons other than VTE prevention due the recent hospitalization for stroke, such as for the secondary prevention of non-AF cardioembolic stroke or dissection, or for atrial fibrillation developed after the index hospitalization (patients with a diagnosis of atrial fibrillation at the time of the index hospitalization were excluded).

In the present study, inpatient prophylaxis with UFH was received by approximately 30% of the ischemic stroke patients, with one in ten patients receiving a LMWH and an eighth receiving mechanical prophylaxis. ACCP guidelines recommend pharmacological prophylaxis with a LMWH or UFH (Grade 1A) for patients with reduced mobility after ischemic stroke [12]. Mechanical prophylaxis with intermittent pneumatic compression or GCS is only recommended for patients with contraindications to pharmacological prophylaxis (Grade 1B). Thigh-length GCS failed to show a significant reduction in the occurrence of symptomatic or asymptomatic proximal DVT compared with avoidance of GCS after acute stroke in the Clots in Legs Or sTockings after Stroke (CLOTS) Trial 1 (10.0% vs. 10.5%, respectively; *P* = 0.88) [21]. However, thigh-length GCS were associated with fewer instances of DVT (6.3%) after acute stroke than with below-knee GCS (8.8%; *P* = 0.008), as observed in the CLOTS Trial 2 [22].In the PREVAIL (PREvention of Venous Thromboembolism After Acute Ischemic Stroke with LMWH and UFH) study of 1762 patients with AIS and restricted mobility, the risk of DVT/PE was 10% following 10 days’ (range 6 to 14 days) prophylaxis with the LMWH enoxaparin, and 18% with UFH (relative risk 0.57; 95% confidence interval 0.44 to 0.76; *P* = 0.0001) [19]. The occurrence of any bleeding complication was similar between groups (both 8%; *P* = 0.83). The composite of symptomatic intracranial and major extracranial hemorrhage was 1% in each group (*P* = 0.23), but there was a slight, clinically significant, excess in major extracranial hemorrhage alone with enoxaparin than UFH (1% vs. 0%; *P* = 0.015).

In the current study, half of the patients who received prophylaxis only received prophylaxis for 1 day—the first day of hospitalization. For those patients who received prophylaxis, the mean ± s.d. prophylaxis duration was 1.9 ± 1.6 days in the inpatient setting and 1.3 ± 5.9 days in the outpatient setting (total 3.1 ± 6.5 days). Several studies have shown reduced VTE risk with extended-duration prophylaxis [3,19,23,24]. In the PREVAIL study, 10.5 ± 3.2 days’ prophylaxis duration was effective at reducing DVT/PE events [19]. Although there are currently no guidelines regarding the most appropriate duration of prophylaxis in stroke patients, there is still a need for sustained use of prophylaxis across the continuum of care i.e. not only while hospitalized, but also post-discharge.

National initiatives including performance measure [25] and financial disincentives [26] have been developed in the US to increase the use of prophylaxis in hospitals in accordance with evidence-based guidelines, and to reduce the clinical and economic burden of VTE. Individual hospitals can also improve the care of ischemic stroke patients by participating in quality initiatives such as the “Get With The Guidelines-Stroke” program, [27] or by implementing ‘standardized stroke orders’ [26]. Standardized stroke orders involve multifaceted interventions based around preprinted discharge orders for stroke patients [28]. After implementation of the stroke orders in six hospitals, optimal DVT prophylaxis within 48 hours significantly increased from 87% at year 1 to 96% by year 2 (*P* = 0.001) [28]. Registries have also been used as tools to define deficiencies and improve quality of care. A voluntary web-based AIS registry was instigated and 50 hospitals reported data on patients diagnosed with ischemic stroke or transient ischemic attack [29]. Rates of optimal DVT prophylaxis within 48 hours among patients with ischemic stroke were found to increase from 76.4% in year 1 to 94.7% by year 4 (*P* = 0.01).

Although the Premier Perspective™-i3 Pharma Informatics linked databases provide real-world information on approximately 275 000 unique patients across the US, there are several limitations to the use of this database for the current study. The database may not be representative of the US ischemic stroke population as a whole with regards to patient age and length of hospitalization. A recent study suggests a trend for decreasing mean age of stroke as shown from 71.2 ± 13.5 years in 1993–1994 to 70.9 ± 14.5 years in 1999, and to 68.4 ± 15.4 in 2005 [30]. Nevertheless, the mean age of our population of patients with a hospitalization for stroke was relatively low (62.2 ± 12.2 years), which could both underestimate the risk of stroke and imply that the patients included in our analysis experienced less severe strokes. Furthermore, the average length of stay of our population (3.0 ± 2.5 days) was shorter than reported previously (5.2 days). This could indicate that patients had recovered mobility quickly following their stroke, and therefore would not have been eligible for thromboprophylaxis after discharge. However, due to the nature of this database analysis, the level of reduced mobility in patients could not be evaluated (either directly or indirectly as a function of length of hospital stay). This limits assessment of actual DVT/PE risk in individual patients, and doesn’t allow the assessment regarding the appropriateness of prophylaxis (considering that the ACCP guidelines only recommend thromboprophylaxis for stroke patients with restricted mobility). Another limitation of this study is that pharmacological prophylaxis alone was assessed in the outpatient setting. Outpatient use of mechanical prophylaxis, such as GCS, was not captured due to over-the-counter availability. Furthermore, the rate of readmission for DVT/PE could be underestimated if patients were readmitted to a hospital that is not included in the database.

## Conclusions

To conclude, this real-world study highlights the clinical burden of DVT and PE in patients with ischemic stroke both in-hospital and post-discharge. DVT prophylaxis was used in 46% of in-hospital patients but only 6% of outpatients. Our study is consistent with potentially inadequate frequency and duration of ‘real-world’ post-stroke thromboprophylaxis.

## Competing interests

Alpesh Amin has received research honorarium and is on the speakers bureau for Sanofi U.S., Inc. Jay Lin is a former employee at Bruce Wong & Associates, Inc., which received funding to carry out this work from Sanofi U.S., Inc. Stephen Thompson is an employee of Sanofi U.S., Inc. Daniel Wiederkehr was at the time of this analysis an employee at Quintiles Consulting, which received funding to carry out this work from Sanofi U.S., Inc. Sanofi U.S., Inc is the manufacturer of enoxaparin (Lovenox®).

## Authors’ contributions

ANA contributed to the study’s conception and design from a clinical perspective and provided clinical expertise in interpreting the analyses; JL and ST participated in the study design, data acquisition, and analysis and interpretation of the data. DW collected the data, conducted statistical analyses, wrote the study report, and participated in interpreting the re’sults. All authors provided input and critical revision of the manuscript, and read and approved the final version for submission.

## Pre-publication history

The pre-publication history for this paper can be accessed here:

http://www.biomedcentral.com/1471-2377/13/17/prepub
